# A Switching Mechanism in Doxorubicin Bioactivation Can Be Exploited to Control Doxorubicin Toxicity

**DOI:** 10.1371/journal.pcbi.1002151

**Published:** 2011-09-15

**Authors:** Nnenna A. Finn, Harry W. Findley, Melissa L. Kemp

**Affiliations:** 1 Wallace H. Coulter Department of Biomedical Engineering, Georgia Institute of Technology and Emory University, Atlanta, Georgia; 2 The Division of Pediatric Hematology/Oncology, Emory University School of Medicine, Atlanta, Georgia; Max-Planck-Institut für Informatik, Germany

## Abstract

Although doxorubicin toxicity in cancer cells is multifactorial, the enzymatic bioactivation of the drug can significantly contribute to its cytotoxicity. Previous research has identified most of the components that comprise the doxorubicin bioactivation network; however, adaptation of the network to changes in doxorubicin treatment or to patient-specific changes in network components is much less understood. To investigate the properties of the coupled reduction/oxidation reactions of the doxorubicin bioactivation network, we analyzed metabolic differences between two patient-derived acute lymphoblastic leukemia (ALL) cell lines exhibiting varied doxorubicin sensitivities. We developed computational models that accurately predicted doxorubicin bioactivation in both ALL cell lines at high and low doxorubicin concentrations. Oxygen-dependent redox cycling promoted superoxide accumulation while NADPH-dependent reductive conversion promoted semiquinone doxorubicin. This fundamental switch in control is observed between doxorubicin sensitive and insensitive ALL cells and between high and low doxorubicin concentrations. We demonstrate that pharmacological intervention strategies can be employed to either enhance or impede doxorubicin cytotoxicity in ALL cells due to the switching that occurs between oxygen-dependent superoxide generation and NADPH-dependent doxorubicin semiquinone formation.

## Author Summary

In the United States, acute lymphoblastic leukemia (ALL) is the most common form of cancer among children. Although the survival rate of childhood leukemia is relatively high, those who do not respond to chemotherapy have very low prognostic outcome. Recent reports point to the critical role of metabolism in determining cell sensitivity to doxorubicin, a conventional drug used in leukemia treatment. Most of the molecular components involved in doxorubicin metabolism have been identified; however, how these components operate as a system and how adaptation of the doxorubicin metabolic network to patient-specific changes in protein components is much less understood. We have therefore chosen to investigate via computational modeling the variations in the distribution of proteins that metabolize doxorubicin can control a cell's ability to respond to doxorubicin treatment. This systems-level approach provides a framework for understanding how patient-specific variability leads to patient-sensitivity to doxorubicin treatment at different doses. With this knowledge, we were able to correctly predict complex behavior induced by pharmacological intervention strategies for manipulation of doxorubicin metabolism. When our interventions are used in combination with doxorubicin, cell viability was promoted or potentiated based on dominant control mechanisms within the metabolic network.

## Introduction

Doxorubicin (Adriamycin, Dox) is an antibiotic anthracycline that is used frequently in chemotherapy for a variety of solid tumors and leukemias [1], [2], [3]. The efficacy of doxorubicin treatment is limited by drug resistance mechanisms [4], [5], [6]. Although the underlying mechanism of doxorubicin resistance is not fully understood, researchers have determined several factors that influence cellular doxorubicin toxicity, most notably the expression of membrane transporters P-glycoprotein/MDR1 (Pgp) [3], [7], [8], [9] and the generation of reactive oxygen species (ROS) and free radicals via doxorubicin redox cycling [10]. Because the modulation of Pgp activity *in vivo* [8], [9] and the use of antioxidants [11], [12] have failed to demonstrate any long term disease-free survival, alternative mechanisms have been proposed to describe the antitumor effects of doxorubicin and thereby offer plausible explanations for why some cancers are sensitive to doxorubicin treatment while others are not.

To this end, the reductive conversion of doxorubicin has been implicated as a major determinant of doxorubicin cytotoxicity and has been proposed as an underlying factor controlling drug resistance in cancer cells [3], [4], [5], [13]. Reductive conversion of doxorubicin is characterized by the one-electron reduction of the quinone moiety of doxorubicin, via NADPH and cytochrome P450 reductase (CPR), into a semiquinone radical [3], [14], [15]. Once the semiquinone radical has been generated, it can exert direct toxic effects or be oxidized back to the quinone form (i.e. redox cycling) [16]. The combination of bioreductive conversion and redox cycling occurs simultaneously in mammalian cells; this overall process is termed bioactivation. It has been reported that the ability of doxorubicin to undergo reductive conversion is dependent on the availability of molecular oxygen and NADPH, and the activities of several intracellular enzymes such as superoxide dismutase (SOD), glutathione peroxidase, NADPH oxidases (NOXs), and thioredoxin [1], [2], [3], [4], [5], [6], [15], components whose intracellular concentrations and activities may vary from one cancer type to the next, or from patient to patient. This variation may help explain some of the contradictory evidence in the literature that describes the proper intracellular environment or intervention strategy for effectively controlling doxorubicin toxicity *in vivo* [4], [5], [6], [12], [16], [17], [18]. For example, doxorubicin-resistant MCF-7 breast cancer cells showed little change in SOD activity compared to their doxorubicin-sensitive counterparts [5]; however, in another study doxorubicin-sensitive MCF cells were rescued via the introduction of SOD [6]. Furthermore, despite the central role of CPR in the bioactivation process, the importance of this enzyme in modulating doxorubicin toxicity has been called into question. While it is widely accepted that CPR is the primary enzyme for catalyzing the reductive conversion of doxorubicin *in vivo* [17], [19], overexpression of CPR does not result in enhanced doxorubicin cytotoxicity [16].

Because the overall network structure for cytosolic doxorubicin bioactivation is believed to be conserved across different cell types [4], [20], [21], the contradictory behavior described above is most likely the result of differences in the intracellular levels of network components (both metabolites and proteins) between cells. *In vitro* studies carried out by Kostrzewa-Nowak et al support this hypothesis by showing that changes in NADPH concentration and SOD activity had a direct impact on degree of doxorubicin reductive conversion [3]. This dependence of the drug on [NADPH] becomes very important in light of recent findings that frequently-occurring somatic mutations in gliomas and leukemias can result in a directional change from NADPH production to NADPH consumption by isocitrate dehydrogenases (IDH1/2) resulting in lower intracellular NADPH levels [22], [23]. Additionally, several lines of evidence in the literature have pointed to the involvement of NOX activity in doxorubicin treatment, providing added relevance to the intracellular levels of NADPH in doxorubicin bioactivation [24]. Thus, the redox context-dependence of doxorubicin metabolism becomes central to accounting for patient variability to anthracycline regimens. Contradictory observations regarding the redox-mediated reactions involved in conferring doxorubicin potency highlight the need for a more in-depth quantitative examination of how the behavior of the doxorubicin bioactivation network is influenced by the initial levels of its system components and its component interactions. The objective of the present study, therefore, was to (a) determine the intracellular factors that control doxorubicin bioactivation for different doxorubicin treatment conditions, (b) develop a mechanistic model of doxorubicin bioactivation in leukemia cells that could be interrogated to predict resistance to doxorubicin treatment prior to clinical administration of the drug, and (c) test, through simulation, the possible intervention strategies that could be employed to modulate doxorubicin cytotoxic activity in leukemia. We exploited previously-published *in vitro* characterization of the biochemical steps involved in doxorubicin bioactivation to develop models that were specific for patient-derived ALL cell lines. Our model findings, confirmed in two cell lines, indicate that doxorubicin metabolism can shift between NADPH-dependent reductive conversion, which drives doxorubicin toxicity in leukemia cells, and NADPH-dependent superoxide generation, which drives doxorubicin-dependent signaling. Nonintuitively, NADPH-dependent ROS production is associated with protection against doxorubicin-induced cell death. Furthermore, redox control over doxorubicin bioactivation is regulated not just by the enzymatic reactions that take place within the cell, but also by the concentration of doxorubicin to which the cell is exposed.

## Results

### A computational model describes *in vitro* doxorubicin bioactivation

**Figure 1:**
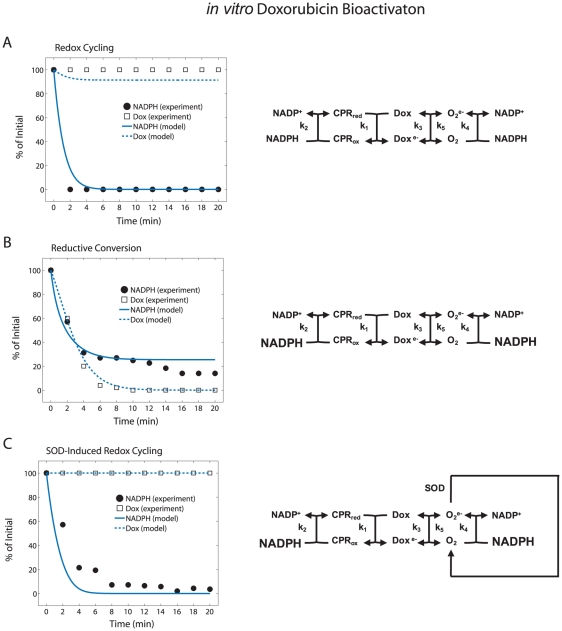
Three proposed mechanisms for *in vitro* doxorubicin bioactivation. (A–C) Experimental data [3] and model fitted results for different doxorubicin bioactivation pathways accompanied by a schematic representation of the hypothesized network underlying each pathway. Large fonts denote experimental conditions in which the [NADPH] was increased from 100 µM to 500 µM.

To investigate the mechanisms that control doxorubicin bioactivation, we developed a kinetic mathematical model of the doxorubicin bioactivation network in a cell free system (Fig. 1). From here on, we shall use the term *in vitro* to refer to acellular systems and the term *in vivo* to refer to cellular systems. Our *in vitro* model was used to reproduce previously published *in vitro* data generated by Kostrzewa-Nowak et al on the effect of NADPH concentration on doxorubicin bioactivation [3]. In the model, we allowed for the reaction of NADPH with molecular oxygen, but assumed it to be non-enzymatic since NADPH oxidase was not present in the cell free reaction mixtures. The inclusion of the NADPH/O_2_ reaction in the bioactivation network model was particularly important because it provided a mechanistic pathway by which increased NADPH concentration could lead to enhanced doxorubicin reductive conversion. Reductive conversion of doxorubicin is characterized by conservative NADPH depletion and quinone doxorubicin transformation, while redox cycling of doxorubicin is characterized by rapid NADPH depletion and sustained quinone doxorubicin. The completed *in vitro* model was capable not only of describing the switch in behavior between reductive conversion and redox cycling of doxorubicin (Fig. 1A, B) based upon the high and low NADPH concentrations, but was also capable of replicating a new experimental condition. Upon inclusion of SOD activity in the bioactivation network, without refitting the parameters, the model demonstrated SOD-induced redox cycling of doxorubicin at high NADPH concentration (Fig. 1C) [3].

### Doxorubicin sensitivity and bioactivation network components differ in EU1 and EU3 ALL cells

**Figure 2:**
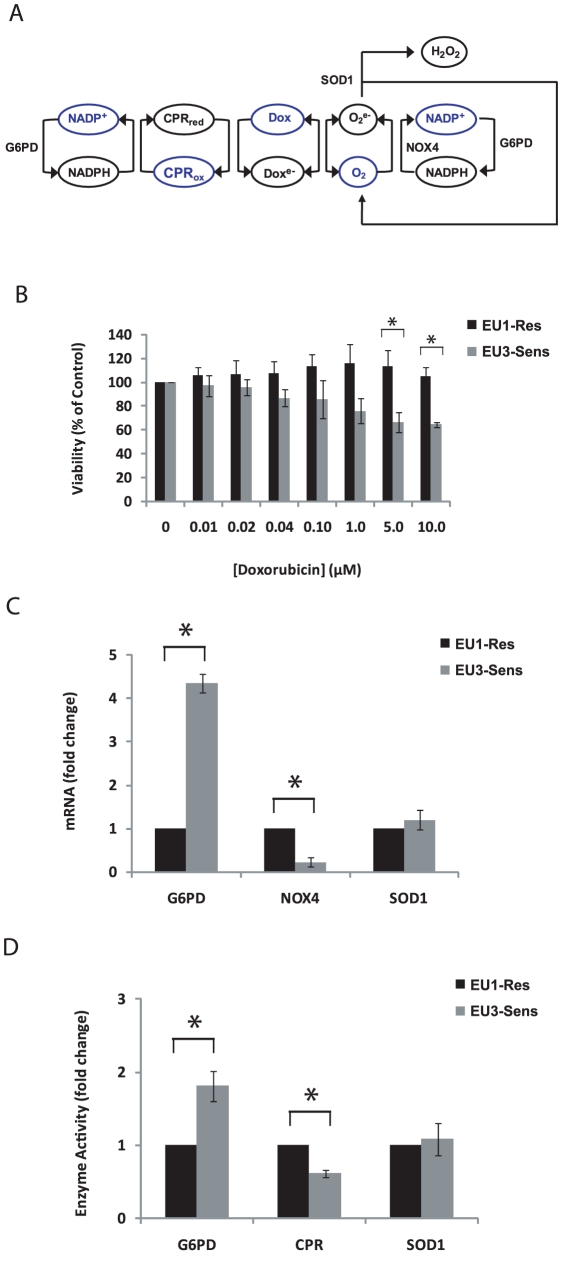
Doxorubicin sensitivity and bioactivation network components differ in EU1 and EU3 ALL cells. (A) Scheme describing *in vivo* doxorubicin bioactivation. (B) Cell viability for EU1-Res and EU3-Sens cells, determined by WST1 assay, after 3 hr doxorubicin treatment at varied concentrations. (C–D) Relative mRNA levels and enzyme activities of enzymes involved in doxorubicin bioactivation in ALL cells. (*p<0.05).

The validated *in vitro* model of doxorubicin bioactivation emphasizes the importance of the reaction between NADPH and molecular oxygen in the accurate representation of doxorubicin bioactivation. Moreover, the model illustrates how the driving force of [NADPH] and levels of SOD can control the switching between reductive conversion and redox cycling. We therefore hypothesized that the intrinsic differences in protein expression and redox state between leukemia cells could similarly give rise to shifts in control between these two processes, conferring differences in doxorubicin cytotoxicity. In support of this hypothesis, others have observed that treatment of the HL60 human leukemia cell line with bioactivated doxorubicin led to increased cytotoxic activity compared to treatment with nonactivated, or redox cycled, doxorubicin [3]. These findings suggest that reductive conversion of doxorubicin may be an important determinant of doxorubicin toxicity in leukemia cells. To further investigate this possibility by computational modeling, we characterized the doxorubicin sensitivity of two ALL cell lines, EU1 (EU1-Res) and EU3 (EU3-Sens), that were previously reported to have over a 10-fold difference in IC50 to doxorubicin [25]. The EU1-Res line displayed limited toxicity to doxorubicin treatment, retaining greater than 100% viability even after exposure to 10 µM of doxorubicin for 3 hrs, whereas the EU3-Sens cell line showed decreased viability after exposure to doxorubicin concentrations as low as 40 nM for the same treatment duration (Fig. 2B).

We characterized the relative mRNA expression levels and activities of the enzymes involved in cytosolic doxorubicin bioactivation (Fig. 2C–D) for these two cell lines. The cellular bioactivation network differs from the *in vitro* one by the inclusion of additional pertinent biochemical reactions (Fig. 2A). Glucose-6-phosphate dehydrogenase (G6PD) enzymatic activity is the primary source for regenerating reduced NADPH in normal metabolism [26] and NADPH oxidases rely on oxygen and NADPH to produce superoxide. It has been previously reported that NOX activity is involved in doxorubicin-induced cell death, implicating NOXs in the cellular doxorubicin bioactivation network [24]. NOX4 is the NADPH oxidase isoform that controls constitutive superoxide production, whereas other isoforms are considered to be activated during signal transduction [27]. The EU1-Res cells contain significantly higher NOX4 mRNA levels and CPR activity, compared to the EU3-Sens cells (p<0.05) (Fig. 2D). EU1-Res cells have significantly lower G6PD mRNA levels (Fig. 2C) and activity (Fig. 2D) (p<0.05). There was no significant difference in the levels of SOD1 mRNA, or SOD1 activity, between the EU1-Res and EU3-Sens cells (Fig. 2C, 2D). There was a direct correlation between mRNA expression and enzyme activity for the enzymes under consideration.

### Cell line specific differences in doxorubicin bioactivation for ALL cells

**Figure 3:**
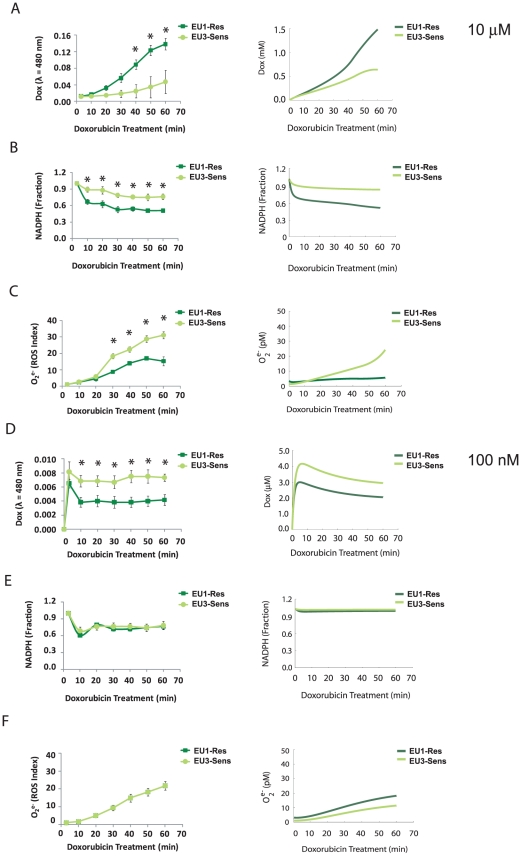
Concentration-dependence of doxorubicin bioactivation in ALL cells. Experimentally-determined and model-predicted quinone doxorubicin accumulation (A), doxorubicin-induced NADPH depletion (B), and doxorubicin-induced superoxide generation (C) in ALL cells treated with 10 µM Dox for 1 hr (*p<0.05). Experimentally-determined and model-predicted quinone doxorubicin accumulation (D), doxorubicin-induced NADPH depletion (E), and doxorubicin-induced superoxide generation (F) in ALL cells treated with 100 nM Dox for 1 hr (*p<0.05).

To examine whether differences in mRNA expression levels and activities of doxorubicin bioactivation enzymes would result in differences in doxorubicin bioactivation between the EU1-Res and EU3-Sens cell lines, we measured intracellular doxorubicin accumulation in the ALL cells for 1 hr during a 10 µM doxorubicin treatment. The EU1-Res cells had significantly higher quinone doxorubicin accumulation compared to the EU3-Sens cells, starting at 40 min of treatment and lasting for the remaining treatment duration (P<0.05) (Fig. 3A). These results were not a function of differential doxorubicin efflux/influx as both the EU1-Res and EU3-Sens cells displayed negligible PgP efflux activity, and the rate of doxorubicin consumption from the cell medium was not significantly different between the cells (Fig. S1, Fig. S2). Because NADPH depletion and superoxide production can be indicators for the extent of doxorubicin reductive conversion that has taken place within a cell [3], we monitored doxorubicin-induced NADPH depletion and superoxide generation in both cell lines. NADPH depletion due to 10 µM doxorubicin treatment was significantly lower in the EU3-Sens cells compared to the EU1-Res cells, starting as early as 10 min into the treatment regimen and continuing this trend for the duration of the treatment (p<0.05) (Fig. 3B). Doxorubicin-induced superoxide generation, measured by HydroCy5, a molecular probe with specificity for ^•^OH and O_2_^•−^ [28], was significantly higher in the EU3-Sens cells than in the EU1-Res cells starting 30 min into the treatment regimen and lasting for the remainder of the treatment duration (p<0.05) (Fig. 3C).

Two *in vivo* models were generated for the EU1-Res and EU3-Sens cells based upon the network structure depicted in Fig. 2A (See Materials and Methods). The differences in quinone doxorubicin accumulation (Fig. 3A) and superoxide generation (Fig. 3C) between the EU1-Res and EU3-Sens cells were accurately captured by the kinetic model simulations. Although kinetic model simulations of doxorubicin-induced NADPH depletion were able to reproduce the depletion trends seen in both the EU1-Res and the EU3-Sens cells, the magnitude of NADPH-depletion in both cell lines was slightly underestimated compared to experimental results (Fig. 3B). Both experimental measurements and model simulations of doxorubicin-induced intracellular doxorubicin accumulation, NADPH depletion, and superoxide generation suggest that the extent of doxorubicin reductive conversion in EU1-Res and EU3-Sens cells differ significantly. The EU1-Res cells exhibited higher quinone doxorubicin accumulation, more NADPH depletion, and lower superoxide generation, which are all consistent with decreased reductive conversion/increased redox cycling, as evidenced by the data generated by our validated *in vitro* model. Conversely, the EU3-Sens cells exhibited lower quinone doxorubicin accumulation, lower doxorubicin-induced NADPH depletion, and higher doxorubicin-induced superoxide generation, which are consistent with the *in vitro* conditions that characterize increased doxorubicin reductive conversion (Fig. 1B, Fig. 3A–C). These results suggest an intrinsic mechanistic switch between redox cycling and reductive conversion that takes place in the EU1-Res and EU3-Sens cells, one that is a function of cell-specific levels of intracellular doxorubicin bioactivation components.

### Concentration-dependence of doxorubicin bioactivation in ALL cells

Because the apparent switch between redox cycling and reductive conversion appeared to be driven by different catalytic rates within the drug metabolism network, we asked whether the concentration of doxorubicin would affect the behavior of the coupled redox reactions. To examine whether differences in the doxorubicin concentration applied to the cells could alter the doxorubicin bioactivation profile of the EU1-Res and EU3-Sens cells, we again analyzed intracellular doxorubicin accumulation, doxorubicin-induced NADPH depletion and doxorubicin-induced superoxide generation in the ALL cells for 1 hr during a 100 nM doxorubicin treatment regimen. The 100 nM doxorubicin concentration represents a 100-fold change in doxorubicin concentration compared to the 10 µM doxorubicin treatment regimen previously administered to the cells. Our experimental results show that the overall shape of the quinone doxorubicin accumulation curve for both ALL cells at the 100 nM doxorubicin treatment level was significantly different that that seen for the 10 µM level. At the 10 µM doxorubicin treatment level, there was a steady increase in the accumulation of quinone doxorubicin in both cell lines as a function of time, although the rate of increase was higher in the EU1-Res cells than the EU3-Sens cells (Fig. 3A). Conversely, at the 100 nM doxorubicin treatment level, there was a rapid increase in quinone doxorubicin accumulation at 10 min, but this increase was followed by a sharp decrease in intracellular quinone doxorubicin which then appeared to equilibrate to a steady state level that was maintained for the rest of the treatment duration (Fig. 3D). Additionally, for the 100 nM doxorubicin treatment regimen, the intracellular quinone doxorubicin levels in the EU1-Res cells were significantly lower than those seen in the EU3-Sens cells (p<0.05) (Fig. 3D), representing a complete switch in behavior compared to that seen at the 10 µM doxorubicin treatment level (Fig. 3A). Without additional parameter fitting, the kinetic simulation of the low doxorubicin treatment condition was able to capture the decreased amounts of quinone doxorubicin observed in the EU1-Res cells, compared to the EU3-Sens cells, as well as the general shape of the intracellular quinone doxorubicin accumulation curve (Fig. 3D), providing further validation of the quality of the cell-line specific models for explaining the complex responses we observed experimentally.

The doxorubicin-induced NADPH depletion in the EU1-Res cells was not significantly different from that seen in the EU3-Sens cells (Fig. 3E). While model simulations accurately predicted similar NADPH depletion trends between EU1-Res and EU3-Sens cells, the underestimation of NADPH depletion in the model simulations was still apparent at the 100 nM doxorubicin concentration condition (Fig. 3E). Differences in doxorubicin-induced superoxide generation between the EU1-Res and EU3-Sens cells were negligible (Fig. 3F) and kinetic model simulations of doxorubicin-induced superoxide generation accurately captured this behavior. The lack of sustained accumulation of quinone doxorubicin in both the EU1-Res and EU3-Sens cells, paired with the experimentally determined NADPH depletion and superoxide generation profiles at the 100 nM doxorubicin treatment condition, suggest that both the EU1 and EU3 cells undergo a shift in the control of their doxorubicin metabolism profiles as a result of changes in the doxorubicin treatment condition applied.

### Model-generated hypotheses of altered NADPH and quinone doxorubicin dynamics are confirmed by pharmacological intervention in drug-sensitive cells

**Figure 4:**
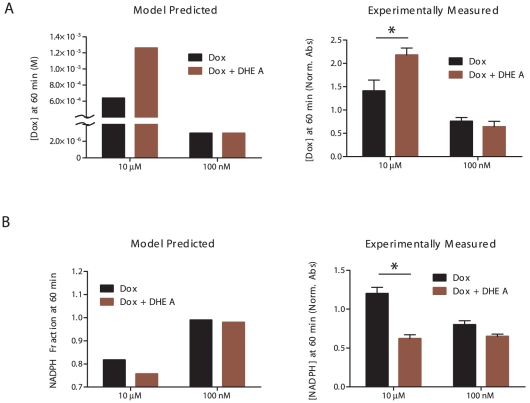
Effects of pharmacological intervention on doxorubicin reductive conversion in EU3-Sens cells. (A) Model-predicted and experimentally determined quinone doxorubicin accumulation in EU3-Sens cells, with and without DHEA intervention, at the 10 µM and 100 nM doxorubicin concentration conditions. (B) Model-predicted and experimentally determined NADPH depletion in EU3-Sens cells, with and without DHEA intervention, at the 10 µM and 100 nM doxorubicin concentration conditions. (DHEA = 10 µM, 24 hrs; *p<0.05).

Concentration-dependent differences in doxorubicin bioactivation exist between the EU1-Res and the EU3-Sens cells (Fig. 3). Based on these differences, we hypothesized that successful intervention strategies for altering the behavior of the doxorubicin bioactivation network within ALL cells would also be doxorubicin concentration-dependent. To test this hypothesis in the EU3-Sens cell line, we conducted a series of pharmacological intervention strategies, for both the 10 µM and the 100 nM doxorubicin concentration condition, that were aimed at decreasing the amount of doxorubicin reductive conversion that occurs within the EU3-Sens cells. We opted to adjust NADPH regeneration (k_8_/k_9_) using the pharmacological G6PD inhibitor, dehydroepiandrosterone (DHEA), because NADPH is involved in the CPR- and oxygen-dependent enzymatic reactions that play a role in reductive conversion and redox cycling of doxorubicin (Fig. 2). Furthermore, simulations of G6PD inhibition on doxorubicin bioactivation in EU3-Sens cells for the 10 µM doxorubicin concentration condition predicted an appreciably increased accumulation of quinone doxorubicin and an increased depletion of NADPH over one hour (Fig. 4A, B). These processes are indicative of increased redox cycling of doxorubicin, at the expense of doxorubicin reductive conversion, and are similar to the dynamics that occur in the doxorubicin-resistant EU1-Res cells (Fig. 3A). Our model predictions were confirmed through pharmacological modification of G6PD activity by the G6PD inhibitor, DHEA, for the 10 µM doxorubicin concentration condition (Fig. 4A, B).

Next, we utilized our kinetic model to simulate the effect of G6PD inhibition on doxorubicin reductive conversion in EU3-Sens cells for the 100 nM doxorubicin concentration condition. Our model predicted that inhibition of G6PD activity in the EU3-Sens cells would have no effect on the accumulation of quinone doxorubicin or the depletion of NADPH over one hour (Fig. 4A, B). Our *in silico* model predictions of the behavior of the doxorubicin bioactivation network after pharmacological intervention at the 100 nM doxorubicin concentration condition were also confirmed (Fig. 4A, B).

### NADPH supply potentially alters viability of doxorubicin-treated ALL cells by controlling semiquinone doxorubicin formation and superoxide generation in a doxorubicin concentration-dependent manner

**Figure 5:**
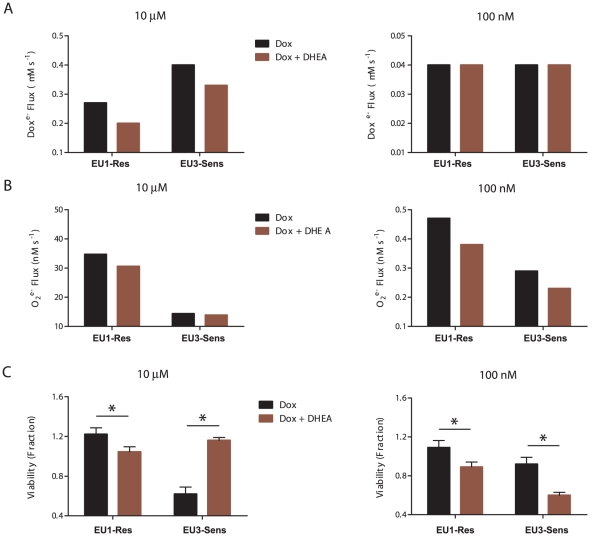
NADPH supply alters doxorubicin sensitivity in ALL cells in a concentration- and cell-dependent manner. (A) *in silico* model predictions of NADPH-dependent semiquinone doxorubicin flux in ALL cells, with and without DHEA intervention, at the 10 µM and 100 nM doxorubicin concentration conditions. (B) *in silico* model predictions of NADPH-dependent superoxide flux in ALL cells, with and without DHEA intervention, at the 10 µM and 100 nM doxorubicin concentration conditions. (C) Experimentally determined (WST1 assay) cell viability for ALL cells after 3 hr doxorubicin treatment, at the 10 µM and 100 nM doxorubicin concentration conditions. (DHEA = 10 µM, 24 hrs; *p<0.05).

To further explore the concentration-dependent effects of DHEA treatment on doxorubicin bioactivation, we used the cellular network models of doxorubicin bioactivation to quantify the fluxes of semiquinone doxorubicin formation and superoxide generation in both the EU1-Res and EU3-Sens cells with and without DHEA treatment. Our analyses suggest that inhibition of NADPH production by G6PD at 10 µM doxorubicin concentration leads to a decrease in the formation of semiquinone doxorubicin in both the EU1-Res and EU3-Sens cells (Fig. 5A), but has no effect on the accumulation of semiquinone doxorubicin in either cell line at the 100 nM doxorubicin condition. Because DHEA will indirectly impact the NADPH-dependent NOX4 by substrate limitations, we also analyzed superoxide fluxes. The models demonstrate that DHEA decreases O_2_^•−^ production in all conditions and cell lines except the EU3-Sens cells at the 10 µM doxorubicin treatment condition (Fig. 5B).

To relate our model findings to experimentally determined changes in cell viability, we analyzed both EU1-Res and EU3-Sens cell survival for the different doxorubicin treatment conditions using a WST1 cell viability assay. Corresponding to our model simulated predictions of quinone doxorubicin accumulation (Fig. 4A), NADPH depletion (Fig. 4B) and semiquinone doxorubicin flux (Fig. 5A), we observed that DHEA was able to rescue EU3-Sens cells from doxorubicin-induced cytotoxicity at the 10 µM doxorubicin concentration condition. Conversely, we found that DHEA treatment at the 10 µM doxorubicin concentration condition significantly decreased cell viability of the EU1-Res cells (p<0.05) (Fig. 5C). At the low doxorubicin concentration condition, DHEA treatment still enhanced doxorubicin toxicity in the EU1-Res cells (Fig. 5C), to a similar degree. However, in the EU3-Sens cells, DHEA treatment at the 100 nM doxorubicin concentration condition enhanced doxorubicin toxicity (Fig. 5C), rather than prevent it.

## Discussion

Although the anthracycline drug doxorubicin is used clinically for the treatment of leukemias and solid tumors [1], [2], [3], the efficacy of doxorubicin treatment is limited by the development of drug resistance [4], [5], [6]. Evidence points to the reductive conversion of doxorubicin as an important ‘first step’ in the regulation of doxorubicin toxicity [2], [3], [4], [5], [13]. While the doxorubicin bioactivation network has been studied extensively, with the overall network structure for cytosolic doxorubicin bioactivation having been deciphered and believed to be conserved across different cell types [4], [20], [21], the adaptation of the bioactivation network to changes in the levels of system components or changes in doxorubicin concentration is much less well understood. Here we show that the doxorubicin bioactivation network is a dynamic system that is sensitive to network component levels and doxorubicin concentrations. Moreover, we illustrate that the intracellular doxorubicin bioactivation network is capable of executing multiple modes of doxorubicin metabolism; the network contains toxicity-generating and ROS-generating reactions that control doxorubicin metabolism via reductive conversion or redox cycling. We illustrate how these reactions can be modulated by pharmacological intervention strategies to either enhance or hinder doxorubicin toxicity in a concentration-dependent manner.

Validation of an *in vitro* doxorubicin bioactivation model reveals that the reaction of molecular oxygen with NADPH is a necessary and significant component of the overall doxorubicin bioactivation network. By analyzing the *in vitro* doxorubicin bioactivation network under the distinctively different conditions described by Kostrzewa-Nowak et al [3], we observed three distinct pathways by which doxorubicin is metabolically altered: CPR-independent redox cycling, CPR-dependent redox cycling, and reductive conversion.

The CPR-independent redox cycling of quinone doxorubicin is the first method by which doxorubicin can be metabolically altered (Fig. 1A). This form of redox cycling of doxorubicin dominates when NADPH is limited. The *in vitro* system has no way of recycling oxidized NADPH once it has reacted with oxidized CPR; when reduced NADPH has been fully consumed, the reduction of quinone doxorubicin by CPR can no longer take place. At this point, the only reactions that can occur are the oxygen-dependent redox cycling reactions of doxorubicin (k_3_/k_5_), which result in a zero net transformation of the quinone doxorubicin molecule and the generation of superoxide.

The second doxorubicin metabolic pathway to consider is the CPR-dependent redox cycling of doxorubicin. CPR-dependent redox cycling of doxorubicin is very similar to CPR-independent redox cycling of doxorubicin in that there is a zero net transformation of quinone doxorubicin into its semiquinone form (Fig. 1C). However, whereas CPR-independent redox cycling takes place at low [NADPH] conditions, CPR-dependent redox cycling takes place when high concentrations of NADPH and molecular oxygen are present simultaneously. When these two conditions are met, the rapid reduction of quinone doxorubicin via CPR occurs, maintained by the high levels of NADPH in the system; the rapid reoxidation of semiquinone doxorubicin by molecular oxygen also occurs, maintained by the SOD-dependent regeneration of molecular oxygen. The analogous *in vivo* scenario was observed in both the EU1-Res and EU3-Sens cells at the low doxorubicin concentration condition (Fig. 3D–F). The NADPH fraction for both cell lines was maintained at a nearly constant level due to the non-enzymatic reactions defined by k_3_/k_5_. Superoxide is produced as a byproduct to a significant degree for a 100-fold lower doxorubicin treatment due to CPR-dependent redox cycling.

The third and final doxorubicin metabolic pathway to consider is the reductive conversion of doxorubicin. When the flux of doxorubicin semiquinone production exceeds the flux of doxorubicin semiquinone consumption, there is a net transformation of quinone doxorubicin into its semiquinone form (Fig. 1B). Doxorubicin reductive conversion dominates at the *in vitro* high [NADPH] condition because there is enough NADPH to support the CPR-mediated reduction of quinone doxorubicin, forcing doxorubicin semiquinone production to overwhelm doxorubicin semiquinone consumption by molecular oxygen. Furthermore, the increased NADPH level diminishes oxygen-dependent semiquinone doxorubicin consumption (k_5_) because NADPH effectively competes with semiquinone doxorubicin for molecular oxygen. We observed the dominance of reductive conversion, *in vivo*, with the EU3-Sens cells during the 10 µM doxorubicin treatment regimen (Fig. 3A). This behavior occurred because as the EU3-Sens cells have an increased capacity to reduce oxidized NADPH, as evidenced by their higher G6PD mRNA and activity levels, they can drive a stronger flux through CPR than their EU1-Res counterparts (Fig. 3A).

**Figure 6:**
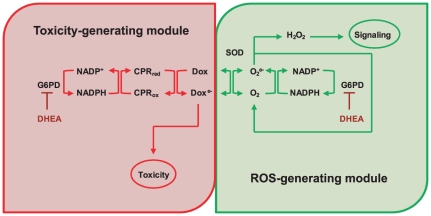
Proposed model of doxorubicin metabolism in ALL cells that emphasizes the toxicity-generating and signal-generating modules comprising the network. The toxicity-generating module is NADPH-limited at the high Dox condition, allowing DHEA administration to decrease NADPH-dependent semiquinone doxorubicin formation. The signal-generating module is NADPH-limited at the low Dox condition, allowing DHEA administration to decrease NADPH-dependent superoxide formation.

After investigating the NADPH-dependent doxorubicin semiquinone and superoxide fluxes that occur during doxorubicin treatment of EU1-Res and EU3-Sens cells, at both the high and the low doxorubicin concentration conditions, and comparing these model generated fluxes to our experimental viability studies (Fig. 5C), we conclude that the doxorubicin bioactivation network is comprised of a toxicity-generating module and a ROS-generating module that likely is implicated in additional signaling (Fig. 6). Our models suggest that at different doxorubicin concentrations, certain components become limiting in either the toxicity-generating module or the ROS-generating module, and these limiting components effectively determine the extent of doxorubicin toxicity that a cell will experience.

Prior *in vitro* biochemical studies have established a minimal concentration of NADPH required to promote the reductive conversion of doxorubicin *in vitro* [3]. We propose that there is a cell-specific set-point of intracellular NADPH availability, as determined by G6PD activity, above which the modulation of NADPH concentration will have little effect on the ROS-generating module of doxorubicin bioactivation within a particular cell. At the high doxorubicin concentration condition, DHEA promoted decreased superoxide flux in the EU1-Res cells, whereas it had little effect on the EU3-Sens cells (Fig. 5B). This is most likely due to the fact that the basal level of NADPH in the EU1-Res cell is already below the threshold level at which the ROS-generating module of doxorubicin bioactivation can be affected by changes in G6PD activity. We have shown experimentally that the basal level of NADPH in the EU1-Res cell is significantly lower than that of the EU3-Sens cell (Fig. S3) making it more susceptible to the effects of DHEA at the high doxorubicin concentration condition, as evidenced by the strong effect of DHEA on cell viability (Fig. 5C). The inhibition of G6PD activity by DHEA at the high doxorubicin concentration condition was able to rescue EU3-Sens cells from doxorubicin induced toxicity because it selectively hindered CPR-dependent doxorubicin reductive conversion (Fig. 5A–C) without affecting the ROS-generating module of doxorubicin bioactivation; the threshold of NADPH below which the ROS-generating module becomes compromised had not yet been reached in the EU3-Sens cells.

Inhibition of G6PD at the low doxorubicin concentration condition did not rescue any of the ALL cells from doxorubicin toxicity, but rather promoted doxorubicin-induced cell death. Because doxorubicin has been shown to activate NOXs *in vivo* [24], NOX activity can be thought of as being dependent on [NADPH], [O_2_], and [Dox]. Therefore, at the low doxorubicin concentration, compared to high, more NADPH is needed to maintain the same level of NOX activity; this effectively lowers the NADPH threshold of the signal generating module. The NOX reaction becomes more sensitive to [NADPH] at the low doxorubicin condition and DHEA can effectively decrease NOX-induced superoxide flux for both cell lines (Fig. 5C). Inspection of the trends between the model fluxes (Fig. 5A–B) and the resultant cytotoxicity (Fig. 5C) suggests that perturbation of the bioactivation network by DHEA affects the CPR-driven reductive conversion component (red module, Fig. 6) at 10 µM doxorubicin and the ROS-producing redox cycling component (green module, Fig. 6) at 100 nM doxorubicin.

It has already been shown in the literature that doxorubicin reductive conversion increases doxorubicin toxicity in cancer cells [3], [17] and our findings corroborate this understanding. When we related our experimental viability studies with our model-simulated flux analyses for the EU1-Res and EU3-Sens cells, a distinct pattern emerged: conditions that hindered the toxicity-generating module of doxorubicin bioactivation decreased doxorubicin-sensitivity, while conditions that hindered the ROS-generating module of doxorubicin bioactivation increased doxorubicin-sensitivity. Moreover, cell-specific levels of NADPH, and to some extent the cell-specific activities of G6PD, determined the ultimate effect of G6PD pharmaceutical perturbation on cell viability at each doxorubicin condition investigated. Therefore, during doxorubicin treatment, one can assume that both the toxicity- and the ROS-generating modules of doxorubicin bioactivation are functioning within a given cancer cell. It is the relative dominance of either the toxicity- or the ROS-generating modules of doxorubicin bioactivation that will ultimately determine cell sensitivity to doxorubicin treatment. A systemic approach to understanding how variability in enzyme activity and concentration control both the toxicity- and the ROS-generating modules of the doxorubicin bioactivation network may provide more efficacious strategies for cancer chemotherapy [29].

We have shown that by limiting the influence of the ROS-generating module of doxorubicin bioactivation, we can effectively promote doxorubicin-induced toxicity in the EU1-Res cell line (Fig. 5), whereas previously it was resistant to doxorubicin treatment (Fig. 2B). Based on these results, it is possible that doxorubicin-induced NOX-dependent ROS generation in the ALL lines serves as a second messenger for downstream signaling pathways that contribute to cell viability. The idea of ROS modulating cell viability is not unprecedented as several intracellular signaling pathways are known to be redox sensitive, the most notable being the NF-κB pathway [30]. The transcription factor NF-κB itself is a redox-sensitive protein [31], [32], [33] known to potentiate cell survival during chemotherapy treatment [34], [35], [36], [37]. Thus, the resulting effect of ROS generation on cell viability most likely involves other downstream signaling pathways.

We have shown that concentration-dependence of doxorubicin bioactivation exists in leukemia cells, with oxygen-dependent, ROS-generating reactions having greater influence over doxorubicin toxicity at low doxorubicin concentrations. If this concentration-dependence is exhibited by a variety of other transformed or non-transformed cells, it could help explain the conflicting evidence in the literature regarding the importance of different enzymatic systems in conferring doxorubicin sensitivity [4], [5], [6], [12], [16], [17], [18]. Work conducted by Asmis et al seems to support the universality of our findings. They observed in macrophages that at low doxorubicin concentrations (0–2 µM) there is a concentration-dependent decrease in the ratio of reduced to oxidized glutathione (GSH/GSSG), a marker or increased oxidative stress; however, when doxorubicin concentrations were increased from 2 µM to 5 µM, the GSH/GSSG ratio was recovered [38]. This finding appears to be in line with our conceptual understanding that at low doxorubicin concentrations, the ROS-generating module of doxorubicin bioactivation is more significant than it is at high doxorubicin concentrations, where it gives way to the toxicity-generating module. The ROS-generating module, however, may also be capable of promoting cell injury in some cell lines. In the same study, Asmis et al report that doxorubicin-induced ROS modified glutathione-dependent thiol oxidation in macrophage cells to promote increased cell injury, implicating both glutathione reductase and glutaredoxin enzymes in the management of doxorubicin-induced cell injury [38]. This result suggests that cell-specific antioxidant capacity may ultimately determine whether doxorubicin-induced ROS promotes cell viability, by modifying signaling pathways, or whether it promotes cell death, by inducing cellular damage via a thiol oxidation-based mechanism.

The two cell-line specific models of doxorubicin bioactivation have demonstrated predictive power and have recapitulated the dynamics of the doxorubicin bioactivation network for multiple conditions. The model behavior, however, falls short in explaining the delayed onset of O2^•−^ or the initial drop in NADPH upon doxorubicin treatment. One reason for this model limitation could be our description of the NADPH-dependent NOX4 enzymatic reaction that utilizes NADPH and molecular oxygen to produce superoxide. The reaction of NADPH with molecular oxygen, as a result of NOX4 activity, was modeled as a function of the concentrations of NADPH, molecular oxygen, and intracellular quinone doxorubicin because it has been shown previously in the literature that doxorubicin treatment promotes intracellular NOX activity in other cell types [24]. Although we have incorporated the doxorubicin-dependence of NOX activity in our ALL models, the lack of knowledge on the exact mechanism by which this interaction occurs introduces some uncertainty into the mathematical formulation we utilized to describe this reaction in our model system. However, it should be noted that our modeling analyses do support the idea that without doxorubicin-dependent NOX activation our description of doxorubicin bioactivation was limited in its ability to thoroughly describe the effect of doxorubicin treatment on NADPH utilization and superoxide generation by the cell.

An additional limitation of our *in vivo* models comes from the fact that our models are incomplete in scope. There are multiple mechanisms for anthracycline bioactivation in mammalian cells: the mitochondria-dependent bioactivation of doxorubicin by mitochondrial complex I and NADH [39], [40], and the mitochondria-independent mechanisms of doxorubicin bioactivation by CPR and NADPH [19]. Furthermore, some studies have placed the cytotoxic action of doxorubicin in the nuclear compartment of mammalian cells [41]. As it currently stands, our model only considers cytosolic doxorubicin bioactivation, and is therefore inherently limited. Additionally, our *in vivo* doxorubicin bioactivation network includes species that are involved in a variety of other intracellular reactions which are independent of doxorubicin bioactivation, such as NADPH. NADPH is a metabolite that is used ubiquitously in cells for a variety of redox dependent reactions [42]. Moreover, NADPH-dependent thiol oxidation-based mechanisms may actually contribute to doxorubicin-induced cell injury in some cells [38], thereby providing a link between intracellular thiol-disulfide status and doxorubicin-induced toxicity; a link that was unaccounted for by our model system because of the qualitative nature of the findings.

The ability of the current *in vivo* models to accurately explain the experimental data and predict new conditions does not immediately preclude alternate mechanisms that may be at work. It is entirely possible that mechanisms beyond the scope of these models contribute to the cell-line differences in doxorubicin sensitivity that are exhibited between the EU1-Res and EU3-Sens cells. We have already provided evidence that altered doxorubicin transport may not be a primary cause of the differential doxorubicin-sensitivity that exists between the EU1-Res and the EU3-Sens cell lines (Fig. S1, Fig. S2). However, non-transport related mechanisms such as altered doxorubicin detoxification, altered replication behavior, or altered ROS metabolism could play a significant role in the doxorubicin toxicity profiles exhibited by these cells, and the importance of these alternate mechanisms may emerge upon characterization of additional cell lines.

Doxorubicin detoxification is thought to be mediated by both one- and two-electron pathways of quinone reduction that depend on the activities of cellular reductases and glutathione S-transferases [19], [43], [44], [45]. Cell-to-cell variation in these enzymes could account for differences in cell sensitivity to doxorubicin treatment. Furthermore, since most mammalian xenobiotic detoxification sytems rely on the addition of a glutathione moeity, via glutathione S transferases [43], variations in the glutathione redox potential of these cells could also contribute to the variations in doxorubicin-sensitivity that are exhibited between the two cells. Moreover, if ROS metabolism is a key factor that determines the sensitivity of cancer cells to doxorubicin treatment, as was suggested by the proposed signaling actions of the ROS-generating module, then differences in glutathione redox potential and differences in other NADPH-consuming mechanisms could effectively promote or hinder doxorubicin toxicity in these cells.

Because additional mechanisms of doxorubicin toxicity may exist, the systematic analysis of these alternate mechanisms are necessary to assess their relative importance *in vivo*. To this end, the current descriptions of doxorubicin bioactivation offered by this study can serve as preliminary models to which additional modules can be easily added. For instance, if one wanted to assess the effect of varied ROS buffering capacity or ROS production on doxorubicin sensitivity across different cell lines, one could merge a comprehensive model of ROS buffering in mammalian cells [42] to the current models. In doing so, experimentally-measured cell-specific values of model components can be inserted into these aggregated models to determine how variations in cell components could affect such aspects as the formation of toxic doxorubicin metabolites, or the ROS-mediated posttranslational modifications that can alter intracellular signaling pathways leading to altered cell growth and proliferation. In this way, future modeling efforts can be utilized to test the contributions of redox and non-redox based mechanisms to the overall levels of doxorubicin-sensitivity experienced by a particular cell.

In summary, examining the cytosolic doxorubicin bioactivation pathway from a systems biology perspective has provided insight into the redox-dependent mechanisms that may be responsible for conferring doxorubicin sensitivity in cancer cells. Kinetic modeling of the electron transfer mechanisms demonstrates that the doxorubicin bioactivation pathway is dual natured and dynamic, exhibiting sensitivity to initial levels of system components, as defined by cell specific enzyme levels, as well as doxorubicin concentration conditions. We have shown through mathematical modeling and experimental analysis, that the toxicity-generating module of doxorubicin bioactivation overwhelms the ROS-generating module in the EU3-Sens cell line, whereas the ROS-generating module of doxorubicin bioactivation overwhelms the toxicity-generating module in the EU1-Res cell line. This discrepancy in doxorubicin metabolism between the EU1-Res and EU3-Sens cells determines the effectiveness of pharmacological intervention strategies that are aimed at modifying doxorubicin induced toxicity. The model elucidates an important role for NAPDH supply, as modulated by G6PD activity, in controlling concentration-dependent doxorubicin cytotoxicity in tumor cells. We demonstrate an approach to enhance doxorubicin cytotoxicity via the pharmacological modification of G6PD activity in both the EU1-Res and EU3-Sens leukemia cell lines. We have also demonstrated, however, that this same intervention strategy used in concert with a high dose of doxorubicin or within a cell containing protein expression levels that promote reductive conversion can actually promote cell viability rather than impede it. The dynamic nature of the doxorubicin bioactivation network, and its ability to metabolize doxorubicin via distinctively different modes, allows for the controlled manipulation of the system to either promote cell viability, as would be desired when protecting non-transformed cells from unwanted doxorubicin toxicity, or to promote doxorubicin-induced transformed-cell death. Finally, because the quinone structure of doxorubicin is conserved across the anthracycline drug family, future studies may elucidate similar control mechanisms in the metabolism of other anthracyclines by cancer cells.

## Materials and Methods

### Computational modeling

Ordinary differential equation [46] models of *in vitro* and *in vivo* doxorubicin bioactivation were developed based on the scheme proposed by Kostrzewa-Nowak et al [3]. Here, the term *in vitro* refers to experiments conducted in solution, while the term *in vivo* refers to experiments conducted within living cells. The *in vitro* model, which describes doxorubicin activation in the presence of NADPH and CPR, contains 6 kinetic parameters and 9 ODEs (see Tables 1 and 2) that describe the changes in concentration of 9 compounds that structure the doxorubicin bioactivation network (doxorubicin, metabolites, redox enzymes and reactive oxygen species). The *in vivo* model, which describes doxorubicin activation in the presence of NADPH, CPR, G6PD, SOD1, and NOX4 is an adaptation of the *in vitro* model and contains 10 kinetic parameters and 10 ODEs (see Tables 3 and 4). The *in vitro* and *in vivo* mathematical models developed in this study use mass action kinetics to describe the enzymatic and non-enzymatic reactions that result in the redox cycling and reductive conversion of doxorubicin. The computational models were designed and numerically integrated using MATLAB R2008a (The Mathworks, Inc., Natick, MA, USA).

**Table 1:** Initial concentration values for the species utilized in the *in vitro* Dox model.

*Species*	*Abbreviation*	*Initial Condition (M)*	*Reference*
Reduced CPR	CPR_red_	1.0 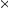 10^−6^	[3]
Oxidized CPR	CPR_ox_	0	Assumption
Quinone Doxorubicin	Dox_q_	1.0 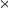 10^−4^	[3]
(SQ) Doxorubicin	Dox_sq_	0	[3]
NADPH	NADPH	1.0 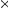 10^−4^/5.0 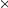 10^−4^	[3]
NADP^+^	NADP^+^	0	Assumption
Molecular Oxygen	O_2_	2.7 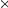 10^−4^	[56]
Superoxide	O_2_^−^	0	Assumption
Hydrogen Peroxide	H_2_O_2_	0	Assumption

**Table 2:** Reaction expressions and parameter values for the *in vitro* Dox model.

*Rxn No.*	*Expression*	*Parameter*	*Reference*
R_1_	k_1_ ([CPR_red_]) ([Dox_q_])	k_1_ = 1.2 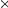 10^4^ M^−1^ s^−1^	Fitted
R_2_	k_2_ ([CPR_ox_]) ([NADPH])	k_2_ = k_1_	[3]
R_3_	k_3_ ([O_2_]) ([Dox_sq_])	k_3_ = 3.0 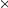 10^8^ M^−1^ s^−1^	[57]
R_4_	k_4_ ([NADPH]) ([O_2_])	k_4_ = 2.9 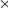 10^1^ M^−1^ s^−1^	Fitted
R_5_	k_5_ ([O_2_^−^]) ([Dox_q_])	k_5_ = 5.5 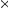 10^7^ M^−1^ s^−1^	Fitted
R_6_	k_6_ ([O_2_^−^]) ([O_2_^−^])	k_6_ = 6.4 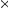 10^9^ M^−1^ s^−1^	[52]

**Table 3:** Initial concentration values for the species utilized in the *in vivo* Dox models.

*Species*	*Abbreviation*	*Initial Condition (M)*	*Reference*
Reduced CPR: (EU1-Res)	CPR_red_	1.3 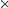 10^−6^	[58]
Reduced CPR: (EU3-Sens)	CPR_red_	8.9 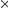 10^−7^	Measured¥
Oxidized CPR	CPR_ox_	0	Assigned
Extracellular (Q) Doxorubicin	Ex_Dox_q_	1.0 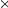 10^−5^/1.0 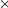 10^−7^	Assigned
Intracellular (Q) Doxorubicin	In_Dox_q_	0	Assigned
Intracellular (SQ) Doxorubicin	In_Dox_sq_	0	Assigned
NADPH: (EU1-Res)	NADPH	3.0 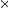 10^−5^	[59]
NADPH: (EU3-Sens)	NADPH	5.4 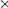 10^−5^	Measured¥
NADP: (EU1-Res)	NADP	NADP = 0.01 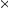 NADPH	[60]
NADP: (EU3-Sens)	NADP	NADP = 0.01 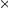 NADPH	[60]
Molecular Oxygen	O_2_	1.5 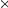 10^−9^	[47], [48]
Superoxide	O_2_^−^	1.5 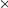 10^−11^	Assigned
Hydrogen Peroxide	H_2_O_2_	1.5 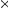 10^−11^	Assigned

**¥:** Measured = Fold change between the resistant and sensitive cell lines (as described in materials and methods) multiplied by the species concentration value for the resistant cell line.

**Table 4:** Reaction expressions and parameter values for the *in vivo* Dox models.

*Rxn No.*	*Expression*	*Parameter*	*Reference*
R_1_	k_1_ ([CPR_red_]) ([Dox_q_])	k_1_ = 1.2 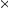 10^4^ M^−1^ s^−1^	*in vitro* model
R_2_	k_2_ ([CPR_ox_]) ([NADPH])	k_2_ = k_1_	*in vitro* model
R_3_	k_3_ ([O_2_]) ([Dox_sq_])	k_3_ = 3.0 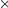 10^5^ M^−1^ s^−1^	[10], [13], [57]
R_4_: (EU1-Res)	k_4_ ([NADPH]) ([O_2_])	k_4_ = 4.2 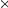 10^4^ M^−1^ s^−1^	[56]
R_4_: (EU3-Sens)	k_4_ ([NADPH]) ([O_2_])	k_4_ = 9.7 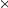 10^3^ M^−1^ s^−1^	Measured¥
R_5_	k_5_ ([O_2_^−^]) ([Dox_q_])	k_5_ = 5.5 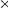 10^7^ M^−1^ s^−1^	*in vitro* model
R_6_	k_6_ ([O_2_^−^]) ([O_2_^−^])	k_6_ = 6.4 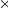 10^9^ M^−1^ s^−1^	*in vitro* model
R_7_: 10 µM	k_7_ ([Ex_Dox_q_]) (A)‡	k_7_ = 1.1 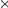 10^−6^ cm s^−1^	Fitted∥
R_7_: 100 nM	k_7_ ([Ex_Dox_q_]) (A)‡	k_7_ = 1.1 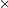 10^−5^ cm s^−1^	[51]
R_8_: (EU1-Res)	k_8_ ([NADP])/( k_9_+[NADP])	k_8_ = 1.8 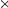 10^−6^ M s^−1^k_9_ = 5.7 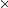 10^−5^ M	Fitted[61]
R_8_: (EU3-Sens)	k_8_ ([NADP])/( k_9_+[NADP])	k_8_ = 3.3 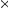 10^−6^ M s^−1^k_9_ = 5.7 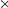 10^−5^ M	Measured¥ [61]

**‡:** A = 10^−3^ (L cm^−3^)
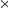
6.15
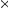
10^−6^ (cm^2^)
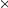
1
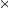
10^9^ (cells/L).

**¥:** Measured = Fold change between the resistant and sensitive cell lines (as determined by basal SOD and G6PD activity) multiplied by the parameter value for the resistant cell line.

**∥:** The permeability constant for doxorubicin permeation is non-constant for the duration of doxorubicin treatment. See Materials and Methods/Text S1 for detailed description.

### Major assumptions of the computational model

To accurately describe the effect of NADPH concentration on the mode of doxorubicin bioactivation that takes place, we allowed the NADPH molecule to react slowly with molecular oxygen in the *in vitro* model. Although this reaction is known to take place *in vivo* through the enzymatic actions of NADPH oxidases [24], due to the high concentration of NADPH contained in the reaction mixture, we assumed the non-enzymatic reaction of NADPH with molecular oxygen could be possible, and as a result, included this reaction at a low rate in the network model of *in vitro* doxorubicin bioactivation. For the *in vivo* kinetic model of doxorubicin bioactivation, we assumed the reaction was catalyzed by NADPH oxidases in a mass action-driven reaction that was dependent on doxorubicin concentration, as it has been shown that doxorubicin treatment can activate NOXs in a doxorubicin concentration-dependent manner [24]. For both the *in vitro* and *in vivo* models, we assumed doxorubicin degradation was negligible within the time period investigated in the study.

The concentration of intracellular molecular oxygen used in the *in vivo* model was derived from literature reported values of oxygen consumption in the HL-60 human leukemia cell line [47]. The rate of oxygen consumption in the HL-60 cell line was reported to be significantly lower than the rate of oxygen consumption in the non-transformed murine macrophage cell line J774A [47], [48]. We used the intracellular oxygen concentration measured for the J774A cell line, in conjunction with the reported oxygen consumption rates for the transformed HL-60 and non-transformed J774A cell lines, to estimate the intracellular concentration of oxygen in the EU1-Res and EU3-Sens lymphoblastic leukemia cell lines [47], [48]. While this may be an inexact estimate of the actual concentration of oxygen in the cell lines being modeled, it does underscore the limited oxygen environment under which cancer cells proliferate [49].

Doxorubicin transport across the cell membrane, as modeled in the *in vivo* models of doxorubicin bioactivation, was described by a concentration gradient multiplied by the permeability constant of doxorubicin. It has been shown previously in the literature that doxorubicin uptake by cells is characterized by a linear diffusive component as well as a saturable, carrier-mediated component [50]. A simplified version of the doxorubicin uptake equation, as presented by El-kareh et al [50], was utilized in the description of doxorubicin bioactivation for the EU1-Res and EU3-Sens cell lines at the high doxorubicin concentration condition. It was assumed that at low doxorubicin concentrations, the saturable, carrier-mediated component of doxorubicin uptake was negligible; therefore for the low doxorubicin concentration condition we utilized a simple diffusion-based equation to describe doxorubicin permeation across the cell membrane [42]. Additionally, it was assumed that the permeability constant for doxorubicin at the low doxorubicin concentration was10× higher than the permeability constant for doxorubicin at the high doxorubicin concentration based on findings by Ghosn et al that illustrated an inverse relationship between solute concentration and solute permeability coefficient [51].

### Parameter fitting

Unknown parameters in the *in vitro* doxorubicin activation model were fitted to *in vitro* experimental data generated by Kostrzewa-Nowak et al. [3]. The fitted parameter values for the *in vitro* model were then used, where applicable, in the *in vivo* doxorubicin bioactivation model and additional parameter fits were made using experimental data generated from doxorubicin-treated ALL cells.

The parameter set of the *in vitro* model contains 6 kinetic parameters and 9 initial conditions. Three of the 6 kinetic parameters that make up the *in vitro* model were fitted to experimentally determined data sets (Table 2). In the fitting procedure, we used the experimental data provided by Kostrzewa-Nowak and colleagues describing the *in vitro* redox cycling and reductive conversion of doxorubicin at varied concentrations of NADPH, doxorubicin, cytochrome P450 reductase (CPR), and superoxide dismutase (SOD) [3]. Because the model is comprised of a simple network with a relatively small number of parameters, parameter fitting was conducted by minimizing the rudimentary cost function, U:
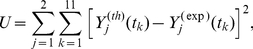
(1)Where 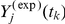 and 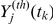 represent the experimental and theoretical (model predicted) data, respectively, of doxorubicin and NADPH (*j* = 1,2), at time points *t_k_* = 0, 2, 4, … , 20 minutes (*k* = 1, 2, … , 11). As an initial approximation of the model parameters to be fitted, we used parameter values estimated from the literature for similar types of enzyme-catalyzed reactions [52], [53]. For fitting purposes, 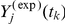 and 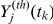 were normalized to their maximal values. All the parameters used in the *in vitro* model are shown in Table 2.

The catalysis of semiquinone doxorubicin was modeled by a two-step process involving first the reduction of doxorubicin by CPR followed by electron transfer by NADPH to oxidized CPR. The reaction rate of reduced CPR with quinone doxorubicin (Reaction R_1_, Table 2) was fitted to the data in [3] for the redox cycling of doxorubicin; the reaction rate for NADPH reacting with molecular oxygen (Reaction R_4_, Table 2) was fitted to experimental data showing the reductive conversion of doxorubicin [3]; the reaction rate for superoxide anion reacting with quinone doxorubicin (Reaction R_5_, Table 2) was fitted to experimental data showing the SOD-induced redox cycling of doxorubicin [3]. The cost function, U, was minimized independently for each fitted parameter because the data used in the fitting procedure was generated from three independent experiments with different sets of initial conditions [3]. The initial conditions for the *in vitro* model were taken directly from the *in vitro* experiments describing redox cycling, reductive conversion, and SOD-induced redox cycling of doxorubicin [3].

The *in vivo* kinetic models of doxorubicin bioactivation were based upon the fitted *in vitro* model of doxorubicin bioactivation that was adapted as indicated in Figure 2A. The parameter set of the model contains 10 kinetic parameters, six of which were either taken directly or estimated from the fitted *in vitro* model, and 10 initial conditions. Two of the 10 kinetic parameters that make up the *in vivo* model had to be fitted to experimentally determined data (Table 4). In the fitting procedure, we used the 10 µM [Dox] NADPH depletion data for the EU1-Res cell line to fit 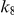, the parameter that describes the rate of NADPH supply by the G6PD enzyme, and we used 10 µM [Dox] extracellular doxorubicin depletion data for the EU1-Res cell line to fit *k_7_*, the parameter that describes the permeability coefficient of doxorubicin (Fig. S2). These parameter fits were conducted for the EU1-Res model only. To determine the fitted parameter value, we minimized the following cost function, U:
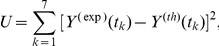

[54] where 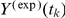 and 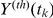 represent the experimental and theoretical (model predicted) data, respectively, of intracellular NADPH or extracellular doxorubicin for the EU1-Res cell line, at time points *t_k_* = 0, 10, …, 60 minutes (*k* = 1, …, 7). As an initial approximation of the model parameter to be fitted, we used parameter values estimated from the literature [42]. For the fitting of parameter *k_8_*, 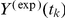 and 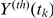 were normalized to their maximal values. Most of the parameters fitted to the EU1-Res experimental data, were used unaltered in the EU3-Sens *in vivo* model. However, to model experimentally determined enzymatic differences between the doxorubicin-resistant EU1-Res cell line and the doxorubicin-sensitive EU3-Sens cell line, we utilized the experimentally determined fold change values between the EU1-Res and EU3-Sens cell lines to estimate appropriate parameter values for the EU3-Sens cell line based on the EU1-Res values previously determined. This method was used to determine the EU3-Res cell line rate constants for NOX4-dependent superoxide generation (*k_4_*), SOD-dependent superoxide dismutation (*k_6_*), as well as G6PD-dependent NADPH reduction (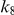).

Because some degree of variation may exist in the values of some of the parameters used in the model, due to limitations in measurement accuracy or due to the inherent differences that exist among *in vivo* cell populations, systematic sensitivity analysis was conducted to determine the extent to which the model predicted results would change as a function of parameter variation (Fig. S4). Details of this sensitivity analysis are highlighted in Text S1.

Tests of pharmacological interventions were conducted *in silico* using the fitted *in vivo* models of doxorubicin bioactivation and assuming 20% inhibition of each target.

### Materials, cell culture and treatment conditions

All reagents were from Sigma-Aldrich unless otherwise specified. Two ALL cell lines representing major phenotypes of childhood acute lymphoblastic leukemia (EU1-Res and EU3-Sens) have been previously characterized [25], [55]. ALL cell lines were cultured in RPM1-1640 medium supplemented with 10% FBS and 100 U/ml of penicillin/streptomycin and grown in a humidified atmosphere of 5% CO_2_ at 37°C. For all experiments, unless otherwise stated, cells were resuspended in fresh media (1
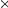
10^6^ cells/ml) and treated with various concentrations of doxorubicin (Enzo Life Sciences), protected from light and incubated at 37°C. Phenol-red-free medium was comprised of phenol-red-free RPMI-1640 medium supplemented with 10% FBS and 100 U/ml of penicillin/streptomycin. For treatments requiring DHEA, ALL cells were incubated in ALL media with the DHEA solution (DHEA in 10% DMSO/90% ALL media) at a final concentration of 10 µM and incubated for 24 hrs prior to dox treatment.

### Cell viability and apoptosis

ALL cells were treated with a range of doxorubicin concentrations for various time periods. After treatment, cell viability was assayed with the cell proliferation reagent WST1 (Roche Applied Science) according to the manufacturer's protocol, using a Synergy 4 hybrid microplate reader (Biotek, Winooski, VT, USA).

### Doxorubicin accumulation

ALL cells plated in 96-well plate format (1
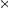
10^6^ cells/ml) were treated with doxorubicin (10 µM or 100 nM) and protected from light at 37°C. Absorbance was read for 1 hr, every 10 min, using a Synergy 4 hybrid microplate reader (Absorbance = 480 nm). The absorbance readings of wells containing media and doxorubicin without any cells, and wells containing cells and media without any doxorubicin, were used as controls.

### NADPH measurement

ALL cells plated in 96-well plate format treated with doxorubicin (10 µM or 100 nM) were protected from light at 37°C. Absorbance was read for 1 hr, every 10 min, using a Synergy 4 hybrid microplate reader (Absorbance = 340 nm). The absorption readings of wells containing media and doxorubicin without any cells, and wells containing cells and media without any doxorubicin, were used as controls. In addition, the absorbance readings of wells containing media and peroxide without any cells, and wells containing media and peroxide with cells, were used as positive controls for NADPH depletion.

### Cellular fractionation and ER isolation

Doxorubicin-treated and untreated cells were pelleted by centrifugation for 5 min at 300
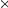
g. Cytoplasmic fractions were obtained by lysing in 2% NP-40 buffer containing 50 mM β-glycerophosphate, 10 mM NaPP, 30 mM NaF, 50 mM Tris-HCL, pH 7.5, 150 mM NaCl, 1 nM benzamidine, 2 nM EGTA, 100 µM sodium orthovanadate, 1 mM DTT, 10 µg/ml aprotinin, 10 µg/ml leupeptin, 1 µg/ml pepstatin, 1 µg/ml microcystin-LR, and 1 mM PMSF. Cells were lysed on ice for 1 hr, followed by centrifugation for 10 min at 14.5
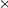
g. For CPR activity analysis, endoplasmic reticulum (ER) isolation from doxorubicin-treated and untreated cells was conducted using the ER isolation kit (Sigma-Aldrich) according to the manufacturer's protocol.

### Enzyme activity measurements

Basal G6PD and CPR activities were determined in EU1-Res and EU3-Sens cells using the Glucose-6-Phosphate Dehydrogenase Assay Kit (BioVision, Mountain View, CA, USA), and the Cytochrome c Reductase (NADPH) Assay Kit (Sigma), respectively, according to the manufacturers' protocols. SOD activity was determined using the Superoxide Dismutase Activity Colorimetric Assay Kit according to the manufacturer's protocol (AbCam).

### qRT PCR measurements

RNA was isolated from cells using the RNeasy isolation kit with RNase-free DNase set according to the manufacturer's protocol. 1 µg of RNA was used for reverse transcription. For detection of mRNA levels, a custom RT2 Profiler PCR Array was used, according to the manufacturer's protocol. The following PCR conditions were used: 10 min at 95°C; 40 cycles of 1 minute at 60°C and 15 seconds at 95°C; melt curve with ramp from 60°C to 95°C. PCR reactions were run using the Applied Biosystems Step One Plus system. Results were normalized to the expression of β-actin. Relative expression levels were calculated using the ΔCT method (2^−ΔCT^). All arrays were performed with triplicate sets of RNA isolation for each cell line for statistical analysis.

### Intracellular ROS determination

For determination of doxorubicin-induced O_2_^•−^ formation, cells were plated at a density of 1
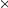
10^6^ cells/ml and pre-incubated with 50 µM Hydro-Cy5 dye [28] resuspended in DMSO for 15 min. After pre-incubation, 10 µM doxorubicin was added to respective wells and kinetic fluorescence readings were taking with the microplate reader every 10 min for 1 hr (Ex = 635 nm, Em = 660 nm). Unstimulated cells, pre-incubated with and without Hydro-Cy5 dye, and phenol red-free media, pre-incubated with and without Hydro-Cy5 dye and doxorubicin, respectively, were used as controls.

### Statistical analysis

All values reported are the average of three or more independent biological replicates +/− standard error. Statistical significance is based upon the criteria of p<0.05 for a Student's t-test (two-tailed, equal variance).

## Supporting Information

Figure S1 **PgP activity in the EU1 and EU3 cells are equivalent and non-significant.** Dye efflux characterization for ALL and AML cell lines indicating that the doxorubicin-resistant EU1 cells and the doxorubicin-sensitive EU3 cells are not significantly different, regarding their PgP activities, from the PgP- AML cell line. (*p<0.05).
(EPS)

Figure S2 **Doxorubicin transport for EU1 and EU3 cells are equivalent.** Extracellular doxorubicin depletion for doxorubicin-resistant EU1 and doxorubicin-sensitive EU3 cells. ([Dox] = 10 µM for 1 hr; *p<0.05).
(EPS)

Figure S3 **Basal NADPH levels are significantly different between the EU1 and EU3 cells.** Relative basal intracellular [NADPH] in doxorubicin-resistant EU1 and doxorubicin-sensitive EU3 cells determined by absorbance readings. (340 nm; *p<0.05).
(EPS)

Figure S4 **Sensitivity analysis of model parameters and species concentrations.** Selected parameters and species initial conditions were systematically perturbed (±10%) and the model-predicted effects of these variations on quinone doxorubicin accumulation, NADPH depletion, and superoxide production were assessed. The initial values used for the sensitivity analysis, *x*, were taken from the EU1-Res cell model at the 10 µM doxorubicin concentration condition. These values were then increased by 10% (+10%) or decreased by 10% (−10%), independently, and then model simulations were carried out: *k* indicates the parameters for which the kinetic rate constants were varied (G6PD, SOD1, and NOX4) and *[ ]* indicates the parameters for which the initial concentrations were varied (NADPH, CPR, and O_2_). Model sensitivity analysis was conducted for a 10 µM doxorubicin treatment regimen. Normalized sensitivity coefficients (*S_i_*) (See Text S1 for details) were calculated to quantitatively characterize the effect of each parameter perturbation on quinone doxorubicin accumulation, NADPH depletion, and superoxide production, respectively. The normalized sensitivity coefficients are shown in Figure S4.
(EPS)

Text S1 **Material and Methods for supplemental figures S1–S4.**
(DOC)
