# Supplementary material for: A Switching Mechanism in Doxorubicin Bioactivation Can Be Exploited to Control Doxorubicin Toxicity
Source: PLoS Comput Biol. 2011 Sep 15;7(9):e1002151. doi: 10.1371/journal.pcbi.1002151 (PMC3174179; doi:10.1371/journal.pcbi.1002151)
Supplement: Text S1 — Material and Methods for supplemental figures S1–S4. (DOC) [file pcbi.1002151.s005.doc]

**Supplemental Materials and Methods**

**Materials, cell culture and treatment conditions.** Human acute myelogenous leukemia (AML) cell lines exhibiting variable PgP expression and doxorubicin resistance (8226/Pgp-, Dox6/Pgp+, and Dox40/PgP++) were donated by Dr. William Dalton, PhD, MD, of the Moffitt Cancer Center (Tampa, FL, USA) [1]. The AML cell lines were cultured in RPM1-1640 medium supplemented with 10% FBS and 100 U/ml of penicillin/streptomycin and grown in a humidified atmosphere of 5% CO_2_ at 37°C. For all experiments, unless otherwise stated, cells were resuspended in fresh media (1 $\times$ 10^6^ cells/ml) and treated with various concentrations of doxorubicin (Enzo Life Sciences), protected from light and incubated at 37°C. Phenol-red-free medium was comprised of phenol-red-free RPMI-1640 medium supplemented with 10% FBS and 100 U/ml of penicillin/streptomycin.

**Doxorubicin depletion.** After ALL cells were treated with 10 µM doxorubicin, phenol-red free media was removed and analyzed for doxorubicin content using the Synergy 4 hybrid microplate reader (Absorbance = 480 nm). The absorption readings of media from wells containing media and doxorubicin without any cells, and wells containing cells and media without any doxorubicin, were used as controls in the extracellular doxorubicin depletion studies.

**DiOC_2_ dye efflux.** DiOC_2_ [3,3’-Diethyloxacarbocyanine iodide] dye (Anaspec Inc.) efflux studies were conducted on ALL and AML cells. 12 $\times$ 10^6^ ALL and AML cells were independently resuspended in 12 ml fresh ALL cell media and incubated for 2 hrs at 37°C in a humidified atmosphere. After incubation, cells were pelleted by centrifugation for 8 min at 1556 rpm. Supernatant was discarded and 12 ml of fresh media was used to resuspend the cells. 3 ml of the 12 ml were transferred to a new tube to serve as a negative control. To the remaining 9 ml, DiOC_2_ was added to a final concentration of 60 ng/ml. All tubes were incubated for 30 min at 37°C in a water bath, gently agitating the tubes every 5 min to ensure adequate mixing. After 30 min incubation, cells were pelleted by centrifugation for 8 min at 1556 rpm. Supernatant was discarded and cells were resuspended in fresh media (3 ml and 9 ml, respectively). The control tube was placed on ice immediately and the total volume in the other tube was divided into three equal parts and placed in three separate tubes. The first aliquot was placed on ice immediately; this was used for the baseline measurement of DiOC_2_ uptake. The cells in the remaining two tubes were pelleted and resuspended in 9 ml of fresh media and incubated for 90 min at 37°C, to measure dye efflux. After 90 min, cells in all tubes were washed with cold PBS and then pelleted. Cell pellets were resustpended in fresh media (4°C) to a final cell concentration of 1 $\times$ 10^6^ cells/ml. Propidium Iodide was added at a concentration of 1 µg/ml to exclude dead cells. Intracellular dye content was then analyzed by flow cytometry (DiOC_2_: Ex = 488 nm, Em = 500 nm).

**NADPH Measurement.** ALL cells plated in 96-well plate format treated with doxorubicin (10 µM) were protected from light at 37°C. Absorbance was read for 1 hr, every 10 min, at 340 nm using a microplate reader. Absorbance values were normalized to absorbance readings of wells containing cell and media without any doxorubicin, as well as wells containing media and doxorubicin without any cells.

**Sensitivity analysis.** Selected parameters and species initial conditions were systematically perturbed (± 10%) and the model-predicted effects of these variations on quinone doxorubicin accumulation, NADPH depletion, and superoxide production were assessed. The initial values used for the sensitivity analysis, *x*, were taken from the EU1-Res cell model at the 10 µM doxorubicin concentration condition. These values were then increased by 10% (+ 10%) or decreased by 10% (- 10%), independently, and then model simulations were carried out. Kinetic rate constants were varied for: G6PD, SOD1, and NOX4; species initial concentrations were varied for: NADPH, CPR, and O_2_. Model sensitivity analysis was conducted for a 10 µM doxorubicin treatment regimen.

**Sensitivity coefficient determination.** The finite difference approximation (FDM) method was used to calculate the sensitivity coefficient, *s_i,j_*, which defined the difference between the nominal and perturbed solutions offered by the model according to the equation:

*s_i,j_(t)* = $\frac{\partial x_{i}(t)}{\partial\theta_{j}}=\frac{x_{i}\left(\theta_{j}+\Delta\theta_{j},t\right)-x_{i}\left(\theta_{j}-\Delta\theta_{j},t\right)}{\Delta\theta_{j}}$ (S1)

where x*_i_*(θ*_j_* + ∆θ*_j_*, t) represents a measured model output at time t, when a particular parameter had been increased by ∆θ*_j_*, and x*_i_*(θ*_j_* - ∆θ*_j_*, t) represents the value of the same model output at the same time, *t*, when the same parameter had been decreased by ∆θ*_j_*. For the purposes of this study, all parameters were perturbed by 10% of their initial values. As a result of the difference in magnitude between parameters, the absolute sensitivity values obtained from Equation S6 were not accurate measures of relative sensitivity. To calculate the relative sensitivities for a more direct comparison of model response at different states across different parameters, we employed the following normalization method:

$\bar{s}$*_i,j_(t)* = $\frac{\partial x_{i}/x_{i}}{\partial\theta_{j}/\theta_{j}}$ (S7)

From this equation, the normalized sensitivity of a particular model output to perturbations in a particular parameter at time, t, could be determined. All sensitivity analysis calculations were done at t = 60 min and parameter sensitivities were calculated with respect to the concentrations of the experimentally determined model species: quinone doxorubicin, NADPH (Fraction), and superoxide.

1. Dalton WS, Durie BG, Alberts DS, Gerlach JH, Cress AE (1986) Characterization of a new drug-resistant human myeloma cell line that expresses P-glycoprotein. Cancer Res 46: 5125-5130.

**Supplemental Figure Legends**

**S1.** **PgP activity in the EU1 and EU3 cells are equivalent and non-significant.** Dye efflux characterization for ALL and AML cell lines indicating that the doxorubicin-resistant EU1 cells and the doxorubicin-sensitive EU3 cells are not significantly different, regarding their PgP activities, from the PgP- AML cell line. (*p < 0.05)

**S2.** **Doxorubicin transport for EU1 and EU3 cells are equivalent.** Extracellular doxorubicin depletion for doxorubicin-resistant EU1 and doxorubicin-sensitive EU3 cells. ([Dox] = 10 µM for 1 hr; *p < 0.05)

**S3.** **Basal NADPH levels are significantly different between the EU1 and EU3 cells.** Relative basal intracellular [NADPH] in doxorubicin-resistant EU1 and doxorubicin-sensitive EU3 cells determined by absorbance readings. (340 nm; *p < 0.05)

**S4.** **Sensitivity analysis of model parameters and species concentrations.** Selected parameters and species initial conditions were systematically perturbed (± 10%) and the model-predicted effects of these variations on quinone doxorubicin accumulation, NADPH depletion, and superoxide production were assessed. The initial values used for the sensitivity analysis, *x*, were taken from the EU1-Res cell model at the 10 µM doxorubicin concentration condition. These values were then increased by 10% (+ 10%) or decreased by 10% (- 10%), independently, and then model simulations were carried out: *k* indicates the parameters for which the kinetic rate constants were varied (G6PD, SOD1, and NOX4) and *[ ]* indicates the parameters for which the initial concentrations were varied (NADPH, CPR, and O_2_). Model sensitivity analysis was conducted for a 10 µM doxorubicin treatment regimen. Normalized sensitivity coefficients (*S_i_*) (See Text S1 for details) were calculated to quantitatively characterize the effect of each parameter perturbation on quinone doxorubicin accumulation, NADPH depletion, and superoxide production, respectively. The normalized sensitivity coefficients are shown in Figure S4.
